# P04.04. Identifying the evidence gaps in acupuncture, experiences of an international project: good practice in traditional Chinese medicine (GP-TCM)

**DOI:** 10.1186/1472-6882-12-S1-P274

**Published:** 2012-06-12

**Authors:** N Robinson, A Lorenc, W Ding, J Jia, M Bovey, X Wang

**Affiliations:** 1 London South Bank University, London, UK; 2 Capital Medical University, Beijing, China; 3 British Acupuncture Council, London, UK

## Purpose

This project aimed to obtain consensus on acupuncture evidence gaps from European and Chinese practitioners and explores the problems and pitfalls for future clinical trials.

## Methods

An online survey to a purposive sample of acupuncture practitioners in the EU and China in 2010/2011 requested information on demographic characteristics, training and education, acupuncture practice, conditions commonly treated, perceived gaps in the evidence and where research should next focus. Data were analysed using bivariate statistics in SPSS, with content analysis of qualitative data.

## Results

Of the 1,126 responses, 1,020 (559 from EU and 461 from China) were included in analysis. Chinese acupuncturists were more likely to practice in hospitals, and EU practitioners, private practice (p<0.001). EU practitioners reported perceiving significant adverse effects more commonly than Chinese practitioners, 14% vs 3%, respectively (p<0.001). Pain was the most commonly treated condition by EU acupuncturists and neurological conditions by Chinese practitioners. Main priorities for research in the EU were obstetrics/gynaecology (infertility, dysmenorrhoea and menopause); for China it was stroke and facial paralysis. Chinese acupuncturists were less likely to want to participate in future trials (27% vs 64%, p<0.001).

## Conclusion

Acupuncture practice and perception of evidence varied widely between practitioners within the EU and China. This can create difficulties for international projects when trying to obtain clarity on evidence gaps and priorities for future research. This study highlighted issues for conducting international projects. Examples included: differences in training; classifying conditions using both TCM pattern differentiation and Western diagnoses; and different professional structures, identifying representative practitioners. The role of acupuncturists in research is critical, but international multi centre research will also require precise clinical trial standards.

